# The dynamic relationship between cerebellar Purkinje cell simple spikes and the spikelet number of complex spikes

**DOI:** 10.1113/JP272259

**Published:** 2016-07-07

**Authors:** Amelia Burroughs, Andrew K. Wise, Jianqiang Xiao, Conor Houghton, Tianyu Tang, Colleen Y. Suh, Eric J. Lang, Richard Apps, Nadia L. Cerminara

**Affiliations:** ^1^ School of PhysiologyPharmacology and NeuroscienceUniversity of BristolBristolUK; ^2^ Bionics InstituteEast MelbourneVictoriaAustralia; ^3^ Department of Neuroscience and PhysiologyNew York University School of MedicineNew YorkNYUSA; ^4^ Department of Computer ScienceUniversity of BristolBristolUK

## Abstract

**Key points:**

- Purkinje cells are the sole output of the cerebellar cortex and fire two distinct types of action potential: simple spikes and complex spikes.
- Previous studies have mainly considered complex spikes as unitary events, even though the waveform is composed of varying numbers of spikelets.
- The extent to which differences in spikelet number affect simple spike activity (and vice versa) remains unclear.
- We found that complex spikes with greater numbers of spikelets are preceded by higher simple spike firing rates but, following the complex spike, simple spikes are reduced in a manner that is graded with spikelet number.
- This dynamic interaction has important implications for cerebellar information processing, and suggests that complex spike spikelet number may maintain Purkinje cells within their operational range.

**Abstract:**

Purkinje cells are central to cerebellar function because they form the sole output of the cerebellar cortex. They exhibit two distinct types of action potential: simple spikes and complex spikes. It is widely accepted that interaction between these two types of impulse is central to cerebellar cortical information processing. Previous investigations of the interactions between simple spikes and complex spikes have mainly considered complex spikes as unitary events. However, complex spikes are composed of an initial large spike followed by a number of secondary components, termed spikelets. The number of spikelets within individual complex spikes is highly variable and the extent to which differences in complex spike spikelet number affects simple spike activity (and vice versa) remains poorly understood. In anaesthetized adult rats, we have found that Purkinje cells recorded from the posterior lobe vermis and hemisphere have high simple spike firing frequencies that precede complex spikes with greater numbers of spikelets. This finding was also evident in a small sample of Purkinje cells recorded from the posterior lobe hemisphere in awake cats. In addition, complex spikes with a greater number of spikelets were associated with a subsequent reduction in simple spike firing rate. We therefore suggest that one important function of spikelets is the modulation of Purkinje cell simple spike firing frequency, which has implications for controlling cerebellar cortical output and motor learning.

## Key points

- Purkinje cells are the sole output of the cerebellar cortex and fire two distinct types of action potential: simple spikes and complex spikes.
- Previous studies have mainly considered complex spikes as unitary events, even though the waveform is composed of varying numbers of spikelets.
- The extent to which differences in spikelet number affect simple spike activity (and vice versa) remains unclear.
- We found that complex spikes with greater numbers of spikelets are preceded by higher simple spike firing rates but, following the complex spike, simple spikes are reduced in a manner that is graded with spikelet number.
- This dynamic interaction has important implications for cerebellar information processing, and suggests that complex spike spikelet number may maintain Purkinje cells within their operational range.

**Abbreviations**
CS: complex spike
CV2: coefficient of variation
ISI: interspike interval
PETH: peri‐event time histogram
RMS: root mean squared
SNR: signal‐to‐noise ratio
SS: simple spike

## Introduction

Central to all major theories of cerebellar function is the interaction between the two distinct types of discharge by Purkinje cells: the complex spikes and simple spikes. Simple spikes are generated intrinsically (Eccles, 1967; Gähwiler, 1975; Häusser & Clark, 1997; Raman *et al*. 1997; Raman & Bean, 1999) and also by activity in the mossy fibre‐granule cell‐parallel fibre pathway and occur at highly variable rates (∼20–200 Hz) (Armstrong & Rawson, 1979; Jirenhed *et al*. 2013; Chen *et al*. 2016). By contrast, complex spikes are generated by activity in the climbing fibre pathway and only occur at ∼1 Hz. The best characterized interaction between complex spikes and simple spikes is the transient cessation in simple spike activity that immediately follows a complex spike (Granit & Phillips, 1956; Thach, 1967; Bell & Grimm, 1969). However, longer modulatory effects (over hundreds of milliseconds) have also been described (Ebner & Bloedel, 1981; McDevitt *et al*. 1982; Sato *et al*. 1992; Wise *et al*. 2010).

Although most studies consider both simple spikes and complex spikes as unitary events, this is only the case in reality for simple spikes. Complex spikes are composed of an initial spike followed by a variable number of secondary components termed spikelets. These spikelets can produce an intense burst of activity (∼500 Hz) and are therefore capable of signalling events that are distinct from those signalled by simple spikes (Campbell & Hesslow, 1986; Yang & Lisberger, 2014). Whether or not a relationship exists between the number of spikelets in a complex spike and the simple spike activity of the same Purkinje cell is unclear (Mano, 1970; Gilbert, 1976). Gilbert (1976) reported a positive relationship between background simple spike firing rate and spikelet number, whereas Mano (1970) and Warnaar *et al*. (2015) found no relationship between complex spike waveform and the preceding simple spike firing rates. With regard to behaviour, a recent study in monkeys has shown that learning‐related reductions in simple spike activity correlate with the duration of complex spikes during motor learning (Yang & Lisberger, 2014). A complex spike waveform may therefore drive experience‐dependent changes in simple spike activity. However, the opposite is equally possible in that simple spike activity could modulate the complex spike waveform, such that complex spikes provide information regarding the recent history of the Purkinje cell (Servais *et al*. 2004).

Another not mutually exclusive possibility is that complex spikes have a homeostatic function and regulate the intrinsic simple spike activity of Purkinje cells (Colin *et al*. 1980; Montarolo *et al*. 1982; Cerminara & Rawson, 2004). Furthermore, evidence exists suggesting that simple spike activity predicts the timing of a complex spike (Miall *et al*. 1998; Chaumont *et al*. 2013; Witter *et al*. 2013). However, in none of these studies was the complex spike spikelet number considered. Thus, considerable uncertainty remains regarding the relationship between simple spike activity and the number of spikelets elicited during a complex spike. The present *in vivo* study aimed to help clarify this important issue and determine the dynamic interplay between simple spike activity and the number of spikelets within a complex spike. We provide evidence consistent with the possibility that the number of spikelets regulates simple spike firing frequency, keeping Purkinje cells within their operational range.

## Methods

Recordings of Purkinje cells were obtained from two different research laboratories (one in Bristol, UK; the other in New York, NY, USA). The data obtained from both laboratories have been used previously but for different analysis (Wise *et al*. 2010; Xiao *et al*. 2014). The Bristol experiments were performed in accordance with the UK Animals (Scientific Procedures) Act 1986 and were approved by the University of Bristol Animal Welfare and Ethical Review Body. Experimental protocols in New York were approved by the Institutional Animal Care and Use Committees of New York University School of Medicine. In brief, adult male Wistar rats (Bristol, *n = *10, ∼300 g) and female Sprague–Dawley rats (NYU, *n = *10, ∼250 g) were anaesthetized with ketamine (100 mg kg^‐1^) and xylazine (5 mg kg^‐1^ or 8 mg kg^‐1^) i.p., and supplementary doses of anaesthetic were administered as required. The depth of anaesthesia was regularly assessed by a paw pinch to monitor reflex muscle tone. Rectal temperature was maintained at 37°C. To gain access to the cerebellum, animals were placed in a stereotaxic frame, and a craniotomy was performed to expose the posterior lobe of the cerebellum.

### Purkinje cell recordings and peripheral stimulation

#### Bristol experiments

Extracellular single unit Purkinje cell recordings were made with glass insulated tungsten microelectrodes (impedance 2 MΩ; Alpha‐Omega, Nazareth, Israel) from copula pyramidis, the paramedian lobule and crus IIa of the posterior cerebellum. The spontaneous and evoked activity of complex spikes and simple spikes of individual Purkinje cells were obtained in the same recording session. Recordings were bandpass filtered between 0.3 and 5.0 kHz and digitized on‐line (sampling rate, 21 kHz) using a Cambridge Electronic Design (CED, Cambridge, UK) 1401 analogue‐to‐digital converter and Spike2 software (CED).

As well as spontaneous Purkinje cell activity, recordings were obtained in response to peripheral electrical stimulation. Bipolar percutaneous stimulating electrodes were inserted into the contralateral whisker pad and the ipsilateral forelimb, and stimuli were given (single pulse; 0.1 ms duration, 1 Hz) at an intensity sufficient to evoke a small but visible muscle twitch from the stimulated body part.

To determine whether the findings found in the anaesthetized rat were also present in awake animals, a small sample of Purkinje cells (*n = *4) with a sufficiently high signal to noise ratio to reliably discriminate individual complex spikes, their associated spikelets and simple spikes were obtained in the lateral part of crus I in a chronically implanted cat (for surgical and recording details, see Miles *et al*. 2006; Cerminara *et al*. 2009). Sample recordings were obtained when the animal was sitting quietly at rest (length of each recording ∼4 mins, involving typically 150–200 complex spikes). Further details of analysis and spike sorting are provided below.

#### New York experiments

Extracellular single unit Purkinje cell recordings were made in crus II and vermis lobule VIII using glass microelectrodes filled with 2.0 m NaCl solution and mounted on a motorized 3D manipulator (MCL‐3; Lang GmbH & Co. KG, Hüttenberg, Germany). Neural activity was recorded using a multichannel recording system (MultiChannel Systems, Reutlingen, Germany) with a 25 kHz/channel sampling rate, gain of 1000× and band pass filters set at 0.2–8.0 kHz.

### Purkinje cell complex spike and simple spike sorting

#### Bristol experiments

In the case of Purkinje cell recordings in anaesthetized rats, simple spike and complex spike activity were discriminated independently off‐line via a template‐matching algorithm (principal component analysis, PCA, Spike2, CED). To detect the spikelets within the complex spike, a positive or negative threshold crossing was manually adjusted and the template duration altered to ensure the capture of the entire complex spike. PCA was then used to cluster the spikelets.

**Figure 1:**
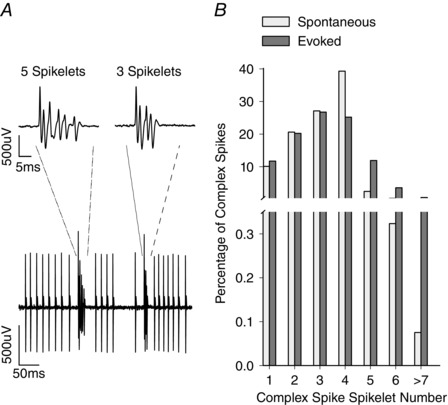
Purkinje cell and spikelet number *A*, example recording from a Purkinje cell showing complex spikes and simple spikes. Two complex spikes are shown with different spikelet numbers occurring close in time. The two complex spikes, representing complex spikes with five and three spikelets, are shown at an expanded time base in the insets. *B*, distribution of spikelet number for spontaneous (white bars) and evoked (grey bars) complex spikes from *n = *61 and *n = *27 Purkinje cells, respectively.

Only Purkinje cell recordings with sufficient signal‐to‐noise ratios (SNRs) to reliably discriminate between secondary spikelets of complex spikes and simple spikes were used in the present analysis (Fig. 1 *A*). SNR was calculated for each individual recording as the mean complex spike amplitude/RMS noise (where RMS = root mean square of the baseline activity when no spiking is present). Only Purkinje cell recordings with a SNR that exceeded 40 were included in the analysis (range 43.2 to 123.4, mean = 78). This SNR is sufficient to distinguish all spikelets from baseline noise. Recordings were also only selected if the peak amplitude of both the simple spikes and complex spikes remained stable throughout the entire recording period (∼60 min, range 17–143 min). The characteristic cessation in simple spike activity following each complex spike (the climbing fibre pause) was used to confirm that recordings were single units and that both types of activity were derived from the same cell (Eccles *et al*. 1966 *b*; Thach, 1967; Sato *et al*. 1992).

For the awake data, the complex spike spikelet number was determined using a Python script (Python Software Foundation. Python Language Reference, version 2.7.7; http://www.python.org) based on a peak detection algorithm that detected the individual time points of the spikelets within the complex spike duration. Simple spikes were discriminated as above.

#### New York experiments

To count spikelet numbers, all complex spikes were high‐pass filtered at 300–400 Hz and automatically processed using a custom‐written procedure (Igor Pro; WaveMetrics Inc., Portland, OR, USA), which detected all deflections with a peak‐to‐peak level exceeding a pre‐defined threshold level.

The resultant spikelet counts for both the Bristol and New York data were manually verified independently by at least two investigators and, on the rare occasion where discrepancies were observed, necessary deletions and/or additions were made.

### Data analysis

Extracted spike and spikelet times were analysed using Python. The initial spike of the complex spike was not included in the spikelet number count because this did not vary across complex spikes. Complex spikes with more than 7 spikelets, although consistent with the general trends found for other spikelet numbers, were excluded from statistical analysis because they accounted for <0.5% of the total complex spike population and were present in only three Purkinje cells and therefore not amenable to statistical analyses.

Complex spikes that occurred within 500 ms of another complex spike were also excluded from our analyses (except in the case of pause duration and interspike intervals; ISI, see below). This was to ensure that any interactions between spikelet number and simple spikes could not be explained by paired pulse depression (which may cause a decrease in the number of spikelets in a subsequent complex spike; Hashimoto & Kano, 1998) or by the modulation of simple spike activity following another complex spike within the time windows tested. Complex spikes that occurred within 0–50 ms after the peripheral stimulation were included in the analysis of evoked responses.

For display purposes, peri‐event time histograms (PETHs; bin width 20 ms) of simple spike firing were constructed around the occurrence of spontaneous or evoked complex spikes with different numbers of spikelets. To aid comparison across all Purkinje cells, histograms were normalized to the number of complex spikes that occurred for each spikelet number and the mean simple spike rate within each Purkinje cell. In all cases, time zero was taken as the onset of the initial spike of the complex spike. Subsequent analysis was performed on raster data rather than on these discretized histograms.

**Figure 2:**
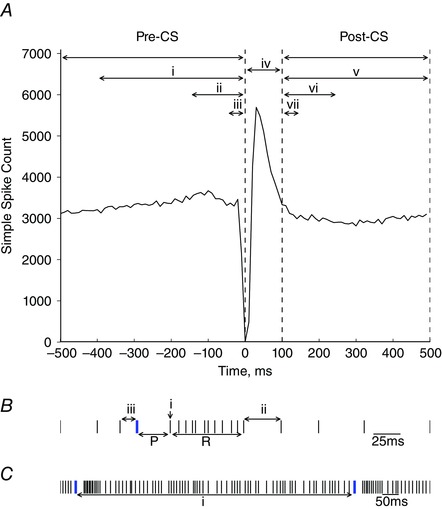
Features of Purkinje cell activity analysed in relation to spikelet number *A*, PETH of simple spike activity around the time of the complex spikes (time = 0 ms). Spikelet number was compared with the simple spike rate before the complex spike in three epochs: (i) –400 to 0 ms; (ii) –150 to 0 ms; and (iii) –50 to 0 ms. (iv) The time interval 0–100 ms after the complex spike that represents the average post‐complex spike pause and rebound in simple spike activity. Spikelet number was also compared with the simple spike rate after the complex spike in three epochs: (v) 100 to 500 ms; (vi) 100 to 250 ms; and (vii) 100 to 150 ms. *B*, schematic raster plot showing simple spikes (black) and complex spikes (blue). Pause duration (P) is calculated as the time from the initial spike in the complex spike to the subsequent simple spike. Rebound duration (R) is defined as the time from the first simple spike after the complex spike (i) to the next simple spike where ISI_SS_ is equal to, or greater than the mean ISI_SS_ (ii). The interspike interval between the simple spike and complex spike (ISI_SS‐CS_) is represented by (iii). *C*, the interspike interval between complex spikes (ISI_CS‐CS_) is illustrated by (i).

To examine the relationship between spikelet number and simple spike activity, specific interactions (Fig. 2) were investigated; first, for each individual Purkinje cell and then for the pooled dataset (Maruta *et al*. 2007).

1. Simple spike rate preceding a complex spike (Pre‐CS): the simple spike rate before the complex spike was calculated as the number of simple spikes in three time epochs: –50 to 0 ms, –150 to 0 ms and –400 to 0 ms before the occurrence of the complex spike at time zero, divided by the duration of the time window (Fig. 2 *A*).
2. Pause duration (P, ISI_CS‐SS_): defined as the time interval between the initial spike of the complex spike and the time of the first simple spike following the complex spike (ISI_CS‐SS_). In 5–10% of cases, another complex spike occurred before simple spike activity resumed and these were excluded from the analysis. To account for the possible influence of simple spike rate on pause duration, pause duration was normalized to mean ISI_SS_/2. In brief, any effect that baseline simple spike firing rate would have on pause duration is excluded by normalizing to the expected pause duration if both simple and complex spike activity were independent. This is equal to half the average ISI_SS_ because, under the independence assumption, a complex spike could fall anywhere between two successive simple spikes with equal probability (Fig. 2 *B*) (Xiao *et al*. 2014).
3. Rebound duration (R): defined as the time from the first simple spike after the complex spike to the simple spike occurring before the ISI_SS_ equal to or greater than the mean ISI_SS_. Purkinje cells that showed either no rebound effect or a heightened simple spike rate that failed to decrease within a 500 ms cut‐off time were excluded from the analysis of rebound duration. Rebound duration is reported in absolute values because no relationship was found with simple spike rate (Fig. 2 *B*).
4. Rebound frequency: calculated as the total number of simple spikes during the rebound divided by rebound duration. Rebound frequencies were normalized to the mean spontaneous simple spike rate observed throughout the spontaneous recording of each Purkinje cell.
5. Post simple spike rate (post‐CS): to quantify the simple spike rate following a complex spike, the number of simple spikes 100 ms after the occurrence of the complex spike within three time epochs (100–150 ms, 100–250 ms and 100–500 ms) was divided by the duration of the time window. The analysis was performed 100 ms after the onset of the complex spike to exclude the average effects of both the pause and rebound (Fig. 2 *A*).
6. The time interval between a complex spike and the preceding simple spike (ISI_SS‐CS_): determined and normalized to the mean ISI_SS_/2, as above for the pause duration (Fig. 2 *B*).
7. Interval between complex spikes (ISI_CS‐CS_): calculated and normalized to the mean ISI_CS_ for each Purkinje cell. Only spontaneous complex spikes were analysed because peripheral stimulation evokes complex spikes with well‐defined latency (Fig. 2 *C*).
8. The change in simple spike rate before and after each individual complex spike: for each complex spike, the simple rate during the –150 to 0 ms time window before the complex spike was subtracted from the simple spike rate during the 100–250 ms time window after the complex spike (which excludes the post‐complex spike pause and rebound). This gave an indication of whether the simple spike rates after individual complex spikes with different numbers of spikelets were lower (negative value), higher (positive value) or the same (0) as before the complex spike.
9. The mean coefficient of variation (CV2) for adjacent ISIs: the regularity of simple spike firing was quantified by determining CV2. This was calculated as CV2 = 2|ISIn + 1 − ISIn|/(ISIn + 1 + ISIn).

### Statistical analysis

All values given in the Results are expressed as the mean ± SD (or the mean ± SEM where indicated), except for pause duration, which was strongly affected by outliers and so the median is given. A two‐tailed unpaired Student's *t* test was used to test for statistical differences between two groups when comparing averages between spontaneous and evoked data. Linear regression analysis was used to assess the relationship between spikelet number and the various parameters tested for individual cells and on pooled data, with the exception of spikelet number *vs*. rebound frequency and time from the preceding simple spike in the evoked conditions, because these relationships were non‐linear and therefore Spearman's rank correlation was used.

## Results

### General characteristics

In the anaesthetized experiments, a total of 27 Purkinje cells from the Bristol dataset and a total of 34 Purkinje cells from the New York dataset met the criteria to be included in our analysis (see Methods). When analysed separately, the two datasets produced similar results; therefore, the data are considered together (*n = *61). The exception was the analysis of evoked activity, which was obtained solely for the Bristol dataset (see below). For all Purkinje cells (average ∼28 min recording duration), none displayed activity related to an injured cell, which typically consists of a progressive reduction over time in simple spike and complex spike amplitudes and abnormally high rates of firing that can be oscillatory in pattern (Eccles *et al*. 1966; Armstrong & Rawson, 1979; Hensbroek *et al*. 2014).

In agreement with previous experiments in awake and anaesthetized preparations (Armstrong & Rawson, 1979; Cerminara & Rawson, 2004; Shin *et al*. 2007; Bosman *et al*. 2010; Rasmussen *et al*. 2014), spontaneous simple spike activity occurred with an average firing frequency of 31.9 ± 17.3 Hz (mean ± SD; range 1.7–70.7 Hz; *n = *61), whereas average complex spike firing rates were 0.89 ± 0.49 Hz (mean ± SD; range 0.15–2.19 Hz; *n = *61). Rhythmic patterns of complex spike activity were only observed in five Purkinje cells (8%) and were not investigated further.

For individual Purkinje cells, the number of secondary spikelets within a given complex spike was highly variable. Overall, the number of spikelets ranged from 0–9, although complex spikes with three or four spikelets were most common (Fig. 1). From the range of spikelets (0–9), there was no statistically significant difference in spikelet number, regardless of whether generated spontaneously or evoked by peripheral stimulation (*P = *0.36, paired Student's *t* test, *n = *27 Purkinje cells, Bristol dataset). Mean spikelet number was also similar for spontaneous (mean ± SD: 3.24 ± 1.36) and evoked (mean ± SD: 3.18 ± 1.40) complex spikes (paired Student's *t* test, *P = *0.06, *n = *27 Purkinje cells, Bristol dataset).There was a significant negative correlation between complex spike firing rate and the number of spikelets generated, with low complex spike rates generating complex spikes with more spikelets (*r* = –0.541, *P* < 0.001, linear regression, *n = *61 Purkinje cells). However, it should be noted that, although Purkinje cells with higher complex spike rates tend to have a lower than average spikelet number, Purkinje cells with a high complex spike rate show the full range of spikelet number and could also elicit a high number of spikelets.

We were unable to find any relationship between complex spikes with different spikelet numbers and the time when they occurred during the recording (i.e. spikelet number was unrelated to whether the complex spike occurred at the beginning, middle or end of the recording period). Taken together with the highly stable and ultra‐low noise recording conditions, we therefore consider it safe to conclude that any systematic differences in spikelet number are most probably not a result of variations in the quality of the recording.

### Relationship between previous simple spike activity and complex spike spikelet number

**Figure 3:**
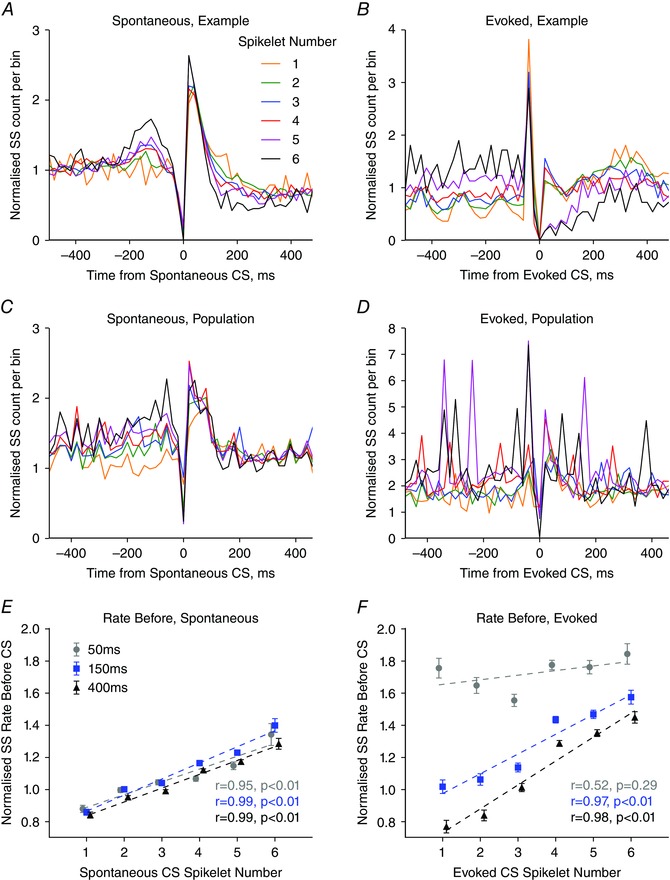
Complex spike spikelet number correlates with simple spike activity *A* and *B*, example simple spike PETH from single Purkinje cell recordings showing simple spike activity around spontaneous (*A*) and evoked (*B*) complex spikes with each number of spikelets. A bin height of 1 represents mean simple spike rate. Bin width = 20 ms. *C* and *D*, as in (*A*) and (*B*) but population average normalized simple spike PETHs for (*C*) spontaneous (*n = *61 Purkinje cells) and (*D*) evoked (*n = *27 Purkinje cells) complex spikes for each spikelet number. *E*, relationship between simple spike rate before the spontaneous complex spikes and spikelet number were positively correlated for the three epochs tested (–50 to 0 ms, grey filled circles; *r* = 0.955, *P = *0.003; –150 to 0 ms blue filled squares; *r* = 0.988, *P < *0.001; –400 to 0 ms, black filled triangles; *r* = 0.993, *P < *0.001, *n = *61 Purkinje cells). *F*, relationship between simple spike rate before the evoked complex spike were positively correlated with spikelet number for two of the three time epochs tested (–50 to 0 ms, grey filled circles; *r* = 0.517, *P = *0.293, –150 to 0 ms blue filled squares; *r* = 0.967, *P = *0.002; –400 to 0 ms, black filled triangles; *r* = 0.981, *P = *0.001, *n = *27 Purkinje cells. Linear regression performed on complex spikes with one to six spikelets. Each data point represents the mean across all complex spikes with that spikelet number. Error bars indicate the SEM.

As a first step in investigating the relationship between complex spike spikelet number and simple spike activity, we analysed spikelet number in relation to the preceding simple spike firing frequency. The different periods of analysis around the complex spikes are shown in Fig. 2 *A*. Example PETHs for two Purkinje cells constructed from spike trains during periods of spontaneous activity and when evoked by peripheral stimulation are shown in Fig. 3 *A* and *B*, respectively. In these two examples, the Purkinje cells discharged complex spikes that varied from one to six spikelets. In both cases, when the spontaneous simple spike firing frequency preceding a complex spike (pre‐CS, Fig. 2 *A*) was greater than the mean firing rate, subsequent complex spikes displayed a greater number of spikelets (linear regression, *P* < 0.001 in both examples).

The largest number of Purkinje cells (34/61; 56% of our sample) showing a significant positive relationship between spikelet number and preceding simple spike frequency was found when the 150 ms epoch was analysed. A possible reason for a subpopulation of Purkinje cells not displaying the effect is provided in the Discussion. Importantly, the relationship between spikelet number and preceding simple spike frequency was also evident when the complex spike data from all Purkinje cells were pooled (*n = *61, Fig. 3 *C*; *n = *27, Fig. 3 *D*). For this reason, the subsequent results relate to population analyses.

There was a strong positive correlation between simple spike activity prior to a spontaneous complex spike and the number of spikelets within the complex spike for all time epochs tested (–50 to 0 ms, grey filled circles; *r* = 0.955, *P = *0.003; –150 to 0 ms blue filled squares; *r* = 0.988, *P* < 0.001; –400 to 0 ms, black filled triangles; *r* = 0.993, *P < *0.001; linear regression, complex spikes with one to six spikelets; Fig. 3 *E*). By contrast, no relationship was found between the variance in simple spike activity before, as measured using the mean CV_2_ value, and spikelet number. Spontaneous complex spikes with only one spikelet were preceded by simple spike rates that were on average 86 ± 2% (*n = *61 Purkinje cells) of the mean simple spike firing frequency. Conversely, complex spikes composed of six spikelets were preceded by simple spike rates that exceeded the mean simple spike firing rate by 135 ± 5% (*n = *61 Purkinje cells). Thus, the number of spikelets in a complex spike correlates positively with preceding simple spike activity.

Similar to the findings for the spontaneous data, a strong positive correlation was also evident between average simple spike rate before an evoked complex spike and spikelet number for two of the three epochs tested (–150 to 0 ms blue filled squares; *r* = 0.967, *P = *0.002; –400 to 0 ms, black filled triangles; *r* = 0.981, *P = *0.001; linear regression, based on complex spikes with one to six spikelets; Fig. 3 *F*). The exception was for the 50 ms time epoch immediately prior to the complex spike (–50 to 0 ms, grey filled circles; *r* = 0.517, *P = *0.293, linear regression, complex spikes with one to six spikelets; Fig. 3 *F*).

### Complex spike and simple spike timing affects spikelet number

**Figure 4:**
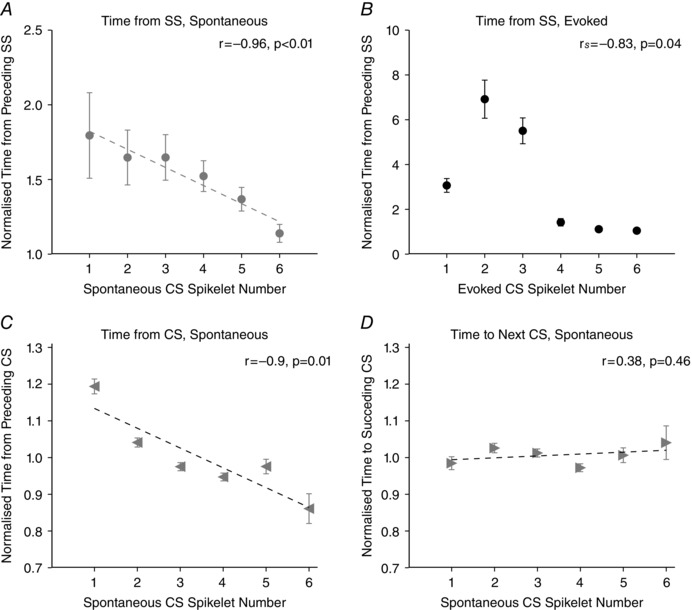
Relationship between number of spikelets in the complex spike and interspike interval of simple and complex spikes *A*, spikelet number of spontaneous complex spikes is inversely related to latency from the preceding simple spike (*r* = –0.965, *P = *0.002, *n = *61 Purkinje cells). *B*, as in (*A*) but for evoked complex spikes (*r*_s_ = –0.829, *P = *0.042, *n = *27 Purkinje cells, Spearman's rank correlation). *C*, spikelet number for spontaneous complex spikes is inversely related to the latency from the preceding complex spike (*r* = –0.903, *P = *0.014, *n = *61 Purkinje cells), although no correlation exists between spikelet number and latency to the subsequent spontaneous complex spike (*D*, *r* = 0.381, *P = *0.457, *n = *61 Purkinje cells). Linear regression analysis and Spearman's rank correlation performed on complex spikes with one to six spikelets for (*A*) and (*B*), respectively. Data are expressed as the mean ± SEM.

The finding that simple spike rate preceding a complex spike is correlated to spikelet number could partly be a result of the timing of the last simple spike prior to the complex spike. This is because there is a greater likelihood that the last simple spike will occur closer in time to the complex spike when firing frequency is high. Consistent with this possibility, spontaneous complex spikes that were preceded by simple spikes closer in time were found to be composed of a greater number of spikelets, which persisted even after normalizing to the mean simple spike rate (*r* = –0.965, *P = *0.002, linear regression, complex spikes with one to six spikelets; Fig. 4 *A*). A negative, non‐linear, correlation was also observed for evoked complex spikes (*r*_s_ = –0.829, *P = *0.042, Spearman's rank correlation, complex spikes with one to six spikelets; Fig. 4 *B*).

**Figure 5:**
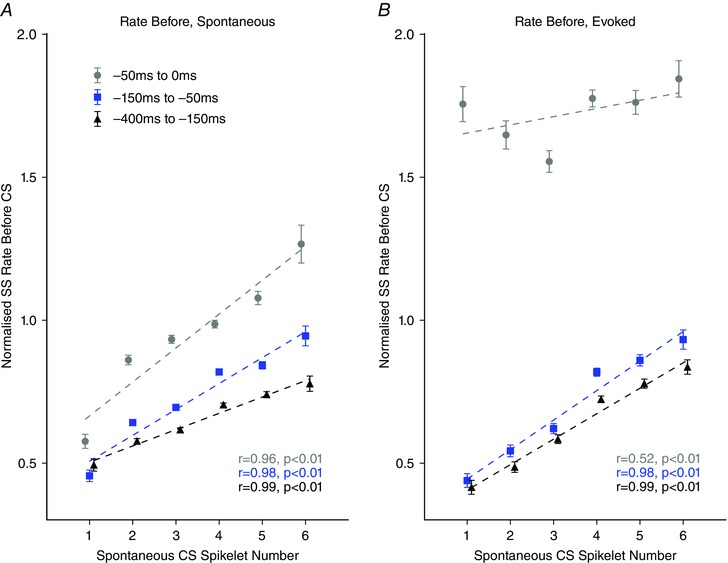
Relationship between simple spike rate before the spontaneous complex spikes and spikelet number for non‐overlapping time windows *A*, relationship between simple spike rate before the spontaneous complex spikes and spikelet number positively correlates for the three epochs tested (–50 to 0 ms, grey filled circles; *r* = 0.963, *P = *0.002; –150 to –50 ms blue filled squares; *r* = 0.976, *P = *0.001; –400 to –150 ms, black filled triangles; *r* = 0.989, *P < *0.001; *n = *27 Purkinje cells). *B*, as in (*A*) but for evoked complex spikes (–50 to 0 ms, grey filled circles; *r* = 0.517, *P = *0.293; –150 to –50 ms blue filled squares; *r* = 0.985, *P < *0.001; –400 to –150 ms, black filled triangles; *r* = 0.990, *P < *0.001; *n = *27 Purkinje cells. Linear regression analysis performed on complex spikes with one to six spikelets. Error bars indicate the SEM.

To establish whether the preceding simple spike rate is correlated with spikelet number rather than the precise timing of the last simple spike, we analysed the simple spike rate preceding complex spikes with each spikelet number for time epochs that did not overlap (–450 to –150 ms, –150 to –50 ms and –50 to 0 ms. We found that a strong positive correlation still exists between simple spike rate prior to the complex spike and spikelet number for each epoch for spontaneous complex spikes (–50 to 0 ms, grey filled circles; *r* = 0.963, *P = *0.002; –150 to –50 ms, blue filled squares; *r* = 0.976, *P = *0.001; –400 to –150 ms, black filled triangles; *r* = 0.989, *P < *0.001; Fig. 5 *A*) and two out of the three epochs for evoked complex spikes (–50 to 0 ms, grey filled circles; *r* = 0.517, *P = *0.293; –150 to –50 ms, blue filled squares; *r* = 0.985, *P < *0.001; –400 to –150 ms, black filled triangles; *r* = 0.990, *P < *0.001; linear regression, complex spikes with one to six spikelets; Fig. 5 *B*). This suggests that the simple spike rate preceding the complex spike is influential to spikelet number.

It is also possible that spikelet number relates to the timing of the preceding complex spike. In this case, there was a negative correlation between the number of spikelets elicited in the complex spike and the latency from the preceding complex spike (*r* = –0.903, *P = *0.014, linear regression, based on complex spikes with one to six spikelets; Fig. 4 *C*). Complex spikes with six spikelets were on average preceded by an ISI_CS_ that was 86% of the mean ISI_CS_, whereas complex spikes with one spikelet tended to be preceded by an ISI_CS_ that was 119% of the mean ISI_CS_. By contrast, the number of spikelets in a given complex spike was not correlated with the timing of the subsequent complex spike (*r* = 0.381, *P = *0.457, linear regression, complex spikes with one to six spikelets; Fig. 4 *D*).

In summary, a number of pre‐complex spike events are correlated with spikelet number, including simple spike frequency and the timing of the preceding simple spike and complex spikes. The previous activity of a Purkinje cell appears therefore to be important in shaping the spikelet number of somatic complex spikes.

### Short‐term interactions between spikelet number and subsequent simple spike activity

Additional analysis examined the relationship between spikelet number and subsequent short‐term changes in simple spike activity. Short‐term interactions were defined as those occurring within 100 ms immediately after a complex spike. This time period includes the post‐complex spike cessation in simple spike activity (pause) and any subsequent transient increase in simple spike activity (rebound). The median pause duration was found to be 68.6 ms (*n = *61 Purkinje cells) following spontaneous complex spikes and 48.4 ms (*n = *27 Purkinje cells) after evoked complex spikes.

No statistically significant correlation was found between the number of spikelets in a complex spike and the duration of the subsequent pause during spontaneous Purkinje cell activity (*r* = 0.746, *P = *0.089, linear regression, complex spikes with one to six spikelets), nor when Purkinje cell activity was evoked by electrical stimulation of the leg or face (*r* = –0.411, *P = *0.419, linear regression, complex spikes with one to six spikelets; data not shown). Thus, even though complex spikes cause the well‐documented pause in simple spike firing, pause duration appears to be independent of the number of spikelets within the complex spike.

A subsequent rebound in simple spike activity (see Methods for definition) was observed in the majority of our sample of Purkinje cells (51/61; 83.6%). Rebound duration varied considerably across Purkinje cells, but on average lasted 77.1 ± 55.4 ms (mean ± SD; range 5.5–362.4 ms; *n = *61 Purkinje cells). The average simple spike rate during the rebound was 82.8 ± 30.1 Hz (mean ± SD; range 30.1–374.27 Hz; *n = *61 Purkinje cells) following spontaneous complex spikes and 70.5 ± 19.9 Hz (mean ± SD; range 27.2–105.8 Hz; *n = *27 Purkinje cells) for evoked complex spikes. No significant correlation was observed between spikelet number and average rebound duration for spontaneous complex spikes (*r* = –0.726, *P = *0.102, linear regression, complex spikes with one to six spikelets; data not shown), nor for evoked complex spikes (*r* = 0.395, *P = *0.439, linear regression, based on complex spikes with one to six spikelets; data not shown).

**Figure 6:**
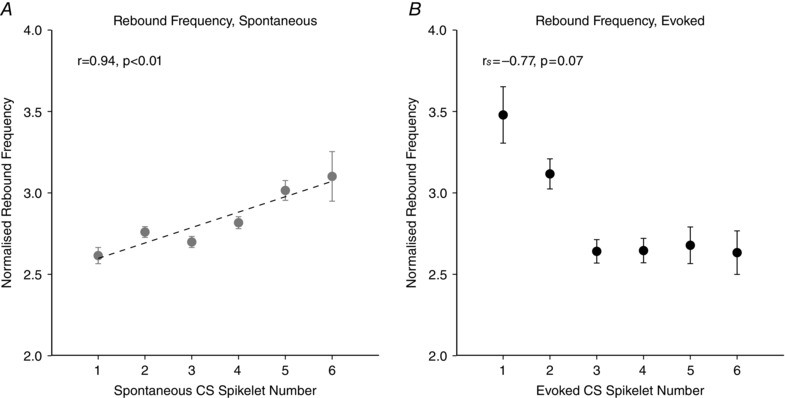
Relationship between spikelet number and frequency of simple spikes during the rebound There is was a significant, positive correlation between spikelet number and the rate of simple spikes in the rebound for spontaneous (*A*, *r* = 0.944, *P = *0.005, *n = *61 Purkinje cells, linear regression) but not evoked complex spikes (*B*, *r*_s_ = –0.771 *P = *0.072, *n = *27 Purkinje cells, Spearman's rank correlation). Analysis performed on complex spikes with one to six spikelets. Data normalized to mean simple spike rate. Error bars indicate the SEM.

However, a statistically significant positive correlation was found between the firing rate of simple spikes during the rebound and spikelet number during spontaneous Purkinje cell activity (*r* = 0.944, *P = *0.005, linear regression, complex spikes with one to six spikelets; Fig. 6 *A*) but not when complex spikes were evoked by peripheral stimulation (*r*_s_ = –0.771 *P = *0.072, Spearman's rank correlation, complex spikes with one to six spikelets; Fig. 6 *B*). This suggests that complex spikes may affect the rate of simple spike firing during the subsequent rebound activity in a manner dependent on the number of spikelets.

### Longer‐term interactions between spikelet number and subsequent simple spike activity

**Figure 7:**
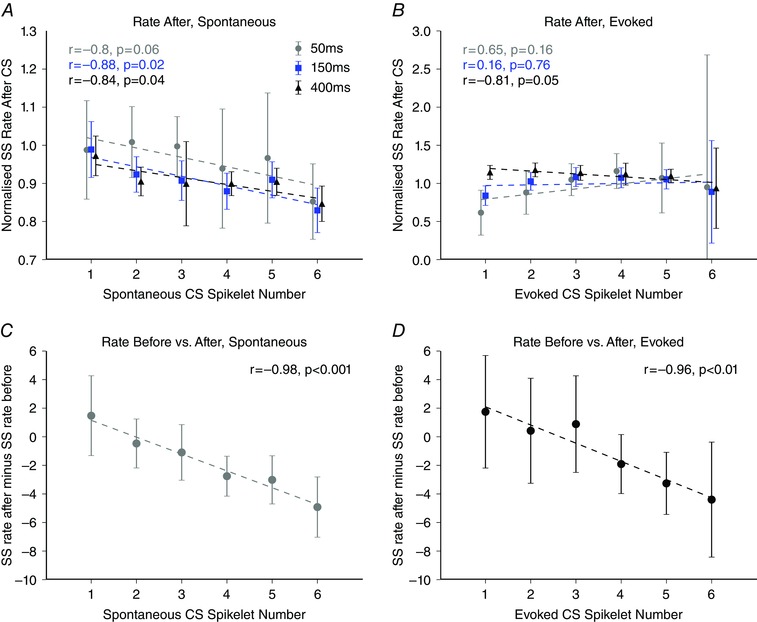
Interactions between spikelet number and simple spike rate after the complex spike *A*, relationship between spikelet number and simple spike rate after spontaneous complex spike events. A negative correlation exists between spontaneous spikelet number and simple spike rate after the complex spike event for two of the three epochs tested (100 to 150 ms, grey filled circles: *r* = –0.800, *P = *0.056; 100 to 250 ms blue filled squares: *r* = –0.881, *P = *0.019; 100 to 500 ms, black filled triangles: *r* = –0.843, *P = *0.035). *B*, as in (*A*) but for evoked complex spikes. No correlation was observed for evoked complex spikes (100 to 150 ms, grey filled circles: *r* = 0.654, *P = *0.159; 100 to 250 ms, blue filled squares: *r* = 0.164, *P = *0.756; 100 to 500 ms, black filled triangles: *r* = –0.812, *P = *0.050). *C* and *D*, magnitude of simple spike depression following a complex spike event was graded with spikelet number for both spontaneous (*r* = –0.984, *P < *0.001, *n = *61 Purkinje cells) and evoked Purkinje cell activity (*r* = –0.964, *P = *0.002, *n = *27 Purkinje cells), respectively. Linear regression analysis performed on complex spikes with one to six spikelets. Error bars indicate the SEM.

We also examined the time period after the post‐complex spike pause and any rebound effects (post‐CS, defined as the time window 100–500 ms after a complex spike, see Methods, Fig. 2). Overall, a significant decrease in average simple spike firing rate was observed following complex spikes (simple spike rate before = 30.1 ± 19.2 Hz, mean ± SD, simple spike rate after = 24.4 ± 16.4 Hz, mean ± SD, *P < *0.001, two‐tailed unpaired Student's *t* test, *n = *20507 complex spikes). A negative correlation was found between spikelet number and simple spike rate following spontaneous complex spike events for time windows >100 ms. This relationship, however, was observed in only 26% of our sample of individual Purkinje cells (16/61) but was evident even when data from all cells were pooled (spontaneous complex spikes; 100–150 ms grey filled circles: *r* = –0.800, *P = *0.056, 100–250 ms blue filled squares: *r* = –0.884, *P = *0.019, 100–500 ms, black filled triangles: *r* = –0.843, *P = *0.035, linear regression, complex spikes with one to six spikelets; Fig. 7 *A*). However, the difference in the simple spike rate following complex spikes with one spikelet compared to six spikelets was modest (∼12%). By comparison, for evoked complex spikes, no correlation was found between spikelet number and simple spike rate after the complex spike for all three time epochs tested (evoked complex spikes; 100–150 ms grey filled circles: *r* = 0.654, *P = *0.159, 100–250 ms blue filled squares: *r* = 0.164, *P = *0.756, 100–500 ms black filled triangles: *r* = –0.812, *P = *0.050, linear regression, based on complex spikes with one to six spikelets; Fig. 7 *B*). Therefore, following evoked complex spikes (and their associated pause and rebound activity) the simple spike rate was not only indistinguishable across complex spikes with different numbers of spikelets, but also from mean simple spike rates. This is in stark contrast to the preceding simple spike rate, which differed systematically with spikelet number for both spontaneous and evoked complex spikes (Fig. 3 *E* and *F*).

The magnitude of simple spike depression following a complex spike event, in comparison with before, appeared to be graded with spikelet number. To test the hypothesis that high simple spike rates prior to a complex spike event are converted to lower rates after the complex spike, in a graded manner depending on spikelet number, we compared the simple spike rate before and after each individual complex spike and related this to spikelet number (see Methods, Fig. 7 *C*). For each individual complex spike, a comparison was made between the rate during the 0 to –150 ms time window before the complex spike and the simple spike rate during the 100–250 ms time window after the complex spike (which excludes the post‐complex spike pause and rebound). This gives an indication of how the simple spike rates vary around complex spike events at the level of individual complex spikes. Spontaneous complex spikes with a greater number of spikelets were associated with subsequent reductions in simple spike firing rate (*r* = –0.984, *P < *0.001, linear regression, complex spikes with one to six spikelets; Fig. 7 *C*). Similarly, when we compared the simple spike rate before and after evoked complex spikes as a function of spikelet number, there was also a decrease in simple spike activity, that increased progressively with increasing numbers of spikelets in the complex spike (*r* = –0.964, *P = *0.002, linear regression, complex spikes with one to six spikelets).

### Data obtained in the awake animal

From a much larger pool of recordings of Purkinje cells obtained from crus I in the awake cat, we determined that the spike trains of four cells had a sufficiently high signal to noise ratio to reliably determine whether any of the interactions found in the anaesthetized preparation were also present in the awake animal. When sitting quietly at rest, simple spike firing rates were on average 40.1 ± 35.9 Hz (mean ± SD; range 5.9–89.3 Hz, *n = *4), whereas complex spike rates were on average 0.77 ± 0.17 Hz (mean ± SD; range 0.57–0.95 Hz, *n = *4). Similar to the much more extensive data obtained in the anaesthetized rat, the number of spikelets in each complex spike in the awake animal ranged from one to eight, with three spikelets being the most common. Also consistent with the data in the anaesthetized rat, a statistically significant positive relationship between pre‐CS simple spike activity and spikelet number was found in two of the Purkinje cells (50%, cell 1, *r* = 0.201, *P = *0.012 *n = *139 complex spikes; cell 2, *r* = 0.160, *P = *0.019, *n = *158 complex spikes; linear regression; for comparison, 56% of Purkinje cells showed such an effect in the anaesthetized preparation). A negative correlation between spikelet number and the simple spike rate after the complex spike was found in one Purkinje cell (25%, *r* = –0.288, *P = *0.001, *n = *189 complex spikes, linear regression; for comparison, 26% of Purkinje cells showed such an effect in the anaesthetized preparation). Despite the small sample, the proportion of cells showing an effect in the awake cat is therefore in remarkably good agreement with the proportions showing the same effect in the anaesthetized rat.

## Discussion

The major findings arising from the present study (Table 1) are that: (i) a strong positive correlation exists between simple spike rate prior to a complex spike and the number of spikelets comprising the complex spike; (ii) spikelet number is related to the timing between the complex spike and the prior occurrence of a simple spike; (iii) spikelet number is positively correlated to the subsequent post‐pause rebound in simple spike rate; and (iv) complex spikes with greater spikelet numbers are followed by simple spike rates that are depressed compared to the rate observed prior to the complex spike event. Thus, the results raise the possibility that spikelet number may be regulating Purkinje cell activity.

**Table 1:** Summary of main findings

	**Spontaneous CSs**	**Evoked CSs**
Preceding SS rate	Positive correlation	Positive correlation
Preceding ISI_SS_	Negative correlation	Negative correlation
Pause duration	None	None
Rebound duration	None	None
Rebound frequency	Positive correlation	None
Following SS rate	Negative correlation	None
Preceding ISI_CS_	Negative correlation	NA
Succeeding ISI_CS_	None	NA

NA, not applicable.

Our findings are in agreement with Gilbert (1976) who found a positive correlation between spikelet number and background simple spike rates in a small population of Purkinje cells located in the anterior lobe of awake monkeys. By contrast, Mano (1970) found no correlation between simple spike rate and spikelet number in Purkinje cells recorded from the oculomotor vermis in awake primates (see also Warnaar *et al*. 2015). This difference is probably not related to species or the effects of anaesthesia because similar findings were found in our small sample of cells from the awake cat.

One possible reason for the discrepancy with the findings of Mano (1970) and Warnaar *et al*. (2015) may be differences in the recording site location, particularly given the emerging evidence that Purkinje cells in zebrin‐positive and zebrin‐negative bands show different firing properties (Paukert *et al*. 2010; Lang *et al*. 2014; Xiao *et al*. 2014; Zhou *et al*. 2014; Cerminara *et al*. 2015). The proportion of cells showing a relationship between simple spikes and spikelet number in the present study may also be related to whether Purkinje cells are located in zebrin‐positive or zebrin‐negative bands.

Regarding whether the number of spikelets can be modulated by previous complex spike activity, Servais *et al*. (2004) found no such relationship, whereas other studies have found that complex spikes preceded by a complex spike occurring closer in time have both greater spikelet numbers (Campbell & Hesslow, 1986) and duration (Warnaar *et al*. 2015). However, the opposite relationship has also been reported (Hashimoto & Kano, 1998; Maruta *et al*. 2007); the difference may be a result of the *in vitro* techniques and analysis methods, respectively. Our findings add to the observation suggesting that, when two complex spikes occur close in time, the second complex spike has a greater number of spikelets.

Although a relationship between spikelet number and preceding simple spike rate was found for spontaneous complex spike activity for all three time epochs investigated, when the complex spikes were evoked by peripheral stimulation, no such relationship was found for simple spikes in the 50 ms time window preceding the complex spikes, nor for simple spike rate in the subsequent rebound. It might be that simple spike activity generated extrinsically, which signals events from the periphery, may require more time to drive changes in cortico‐nucleo‐olivary loops (see below).

### Ionic control of spikelet modulation

Complex spikes are generated as a result of interactions between Na^+^, Ca^2+^ and K^+^ currents (Schmolesky *et al*. 2002; Hurlock *et al*. 2008) with the initiation of the complex spike and its spikelets occurring at the Purkinje cell axosomatic membrane (Zagha *et al*. 2008; Veys *et al*. 2013). Resurgent Na^+^ currents and Kv3.3 currents are critical determinants of the complex spike waveform by underpinning repetitive spikelet generation (Raman *et al*. 1997; Raman & Bean, 1999; Zagha *et al*. 2008; Veys *et al*. 2013). The *I*_h_ current is also important in determining the relationship between complex spike events and simple spike firing patterns (Loewenstein *et al*. 2005). It is possible therefore that the interactions between *I*_h_, Kv3.3 and resurgent sodium currents establish the relationship observed in the present study between complex spike spikelet number and simple spike rates.

Despite the varying simple spike firing frequencies observed prior to complex spikes with varying numbers of spikelets, our findings suggest that the simple spike rate appears to equalize to some extent after complex spikes, especially in the evoked condition where simple spike rates return to mean firing frequencies after complex spikes with each spikelet number despite the variations observed before. Simple spike activity is dependent on a balance between Na^+^ and K^+^ conductances (Llinas & Sugimori, 1980; Raman & Bean, 1997, 1999). Previous studies have shown that climbing fibres can control simple spike firing rate via the activation of Ca^2+^‐dependent K^+^ currents, as triggered by the rise in intracellular Ca^2+^ that occurs with climbing fibre activation (Tank *et al*. 1988; Eilers *et al*. 1995; Womack & Khodakhah, 2004; McKay *et al*. 2007; Rinaldo & Hansel, 2010). Variations in spikelet number could cause fluctuations in the level of intracellular Ca^2+^ and accompanying Ca^2+^‐dependent K^+^ currents, which in turn would govern the rate of Purkinje cell discharge.

### Cerebellar circuit control of spikelet number and Purkinje cell activity

The evidence available to date suggests that the pause in simple spike firing that follows a complex spike, is driven by extrinsic events such as local interneurone activity (Granit and Phillips, 1956; Sato *et al*. 1992; Jörntell and Ekerot, 2003). And evidence indicates that this is driven by climbing fibre connections with molecular layer interneurones (Marshall and Lang, 2009; Mathews *et al*. 2012). In the present study, the number of spikelets was not found to correlate with pause duration; therefore, it follows that the regulation of spikelet number is probably a result of direct olivary network effects on the target Purkinje cells.

One alternative mechanism that could regulate spikelet number is the number of spikes in olivary bursts (Mathy *et al*. 2009; Bazzigaluppi *et al*. 2012), in addition to factors that alter the amplitude and/or phase of olivary subthreshold oscillations (Mathy *et al*. 2009; Bazzigaluppi *et al*. 2012; De Gruijl *et al*. 2012) and the synchronization of complex spikes (Lang *et al*. 2014). The cerebellar nuclei possess a population of inhibitory GABAergic neurones that project to the inferior olive (Andersson *et al*. 1988; Nelson & Mugnaini, 1989); thus, the olivo‐cortico‐nuclear projections form a closed loop, suggesting that simple spike activity, via its action on nucleo‐olivary neurones, could help determine spikelet numbers by altering the state of the inferior olive. Evidence that the simple spike activity of each cortical region does indeed help control its own complex spike activity was first provided as a result of the pharmacological manipulation of simple spike levels, which induced correlated changes in local complex spike firing rates and synchrony levels (Marshall and Lang 2009). Consistent with these findings, optogenetic stimulation of the nucleo‐olivary projection has been shown to cause a dampening or cessation of subthreshold oscillations and a reduction in the coupling of olivary cells (Lefler *et al*. 2014). Moreover, optogenetic activation of Purkinje cells was found to disinhibit the inferior olive, resulting in the subsequent activation of complex spikes with a latency of ∼100 ms (Chaumont *et al*. 2013; Witter *et al*. 2013). Thus, to some extent, Purkinje cell output can control afferent climbing fibre activity and thereby control complex spike spikelet number via the ionic mechanisms outlined above. Consistent with this, we observed a small peak in simple spike activity ∼100 ms prior to spontaneous complex spike events (Fig. 3 *A*). The combination of the current and previous results therefore suggests that cortico‐nucleo‐olivary loops are important in controlling complex spike activity. Moreover, changes in complex spike waveform (and spikelet content specifically) have been correlated with complex spike synchrony levels (Lang *et al*. 2014). Thus, Purkinje cells, via their influence on nucleo‐olivary neurones, may be capable of controlling spikelet number.

Miall *et al*. (1998) proposed that the ongoing activity of parallel fibres would, if left unchecked, lead to an ever increasing level of Purkinje cell simple spike firing. They suggested that complex spike activity in the absence of movement may have an ‘autocorrective’ effect, in that the increasing simple spike activity will increase climbing fibre activity via the cortico‐nuclear‐olivary loop. In support of this, high frequency stimulation of climbing fibres causes a reduction in simple spike firing rates and conversely, olive lesioning and the inhibition of complex spikes results in high rates of simple spike discharge (Colin *et al*. 1980; Rawson & Tilokskulchai, 1982; Cerminara and Rawson, 2004; Bengtsson and Hesslow, 2013). Moreover, variations in simple spike activity (both pharmacological and spontaneous) are correlated with changes in complex spike firing rates (Marshall and Lang, 2009). The present study supports this suggestion, and develops the concept by proposing that spikelet number may be an important determinant of the level of autocorrection. Complex spikes with higher numbers of spikelets were found to depress simple spike rates to a greater degree than those with fewer spikelets.

### Function of spikelets

A variety of different functions have been proposed for the climbing fibre system, including the regulation of simple spike rates, a role in motor timing and a role in motor learning (Simpson *et al*. 1996). With respect to motor learning, complex spikes are assumed to represent an error or teaching signal (Ito, 2001) and recent studies have shown that the type of plasticity induced by complex spikes is dependent on the number of spikelets: a single climbing fibre impulse paired with parallel fibre stimulation results in long‐term potentiation, whereas bursts of climbing fibre impulses in conjunction with parallel fibre stimulation result in long‐term depression (Mathy *et al*. 2009). Graded climbing‐fibre induced calcium signals have also been observed in Purkinje cell dendrites in response to eye‐blink conditioning (Najafi *et al*. 2014), which may have implications for short‐ and long‐term plasticity. Indeed, behavioural studies have also shown that, during learning of smooth pursuit eye movements, simple spike activity undergoes trial‐by‐trial depression that is related to the duration of complex spikes (Yang & Lisberger, 2014). Furthermore, bursts of climbing fibre stimulation have been shown to result in the acquisition of Purkinje cell conditioned responses during eye blink conditioning, whereas single climbing fibre impulses cause extinction of the previously acquired response (Rasmussen *et al*. 2013). Taken together, these previous findings therefore suggest that spikelet number appears to be closely linked to learning processes in the cerebellum.

However, as outlined above, another possible function of complex spikes may be to regulate cerebellar cortical activity. Previous investigations have found that simple spikes evoked by peripheral sensory stimulation after the occurrence of a complex spike exhibit a short lasting (∼200 ms) enhancement in their responsiveness (Ebner *et al*. 1983; Ebner & Bloedel, 1984). Furthermore, Rawson and Tiloskulchai (1982) found that the intrinsic simple spike activity of Purkinje cells is suppressed by repetitive stimulation of climbing fibres, whereas simple spike responses evoked by parallel fibre stimulation were not. Taken together, these studies suggest that complex spikes produce a ‘gain change’ in simple spike activity to incoming signals (the gain change hypothesis; Ebner and Bloedel, 1981). The present study adds the possibility that spikelet number plays a role in determining simple spike activity levels. We found that simple spike rate during the rebound positively correlates with spikelet number; thus, complex spikes with greater numbers of spikelets result in higher transient increases in gain. This is followed by a subsequent return or a modest undershoot to baseline levels of simple spike activity. Spikelets could also lead to a reduction in intrinsic simple spike discharge such that extrinsic activity is accentuated. This is in line with recent studies indicating that complex spikes may have the capacity to multiplex (Ohmae & Medina, 2015); the number of spikelets in a complex spike may maintain Purkinje cells within their operational range at the same time as signalling extrinsic events related to cerebellar learning.

## Additional information

### Competing interests

The authors declare that they have no competing interests.

### Funding

This work was supported by the MRC (RA, NLC), Wellcome Trust (AB, AKW, CH, RA) and the National Science Foundation (JX, TT, CH, EJL).

### Author contributions

Experiments were performed at The University of Bristol and New York University. EJL, RA and NLC were responsible for the study design. AKW, JX, TT, EJL and NLC carried out the experiments. AB, JX, TT, CH, TT, CYS, EJL, RA and NLC analysed and interpreted the data. AB, RA and NLC drafted the paper. All authors revised the article critically for important intellectual content. All authors have approved the final version of the manuscript and agree to be accountable for all aspects of the work. All persons designated as authors qualify for authorship, and all those who qualify for authorship are listed.
